# The life experience of leprosy families in maintaining interaction patterns in the family to support healing in leprosy patients in Indonesian society. A phenomenological qualitative study

**DOI:** 10.1371/journal.pntd.0010264

**Published:** 2022-04-08

**Authors:** Abd Nasir, Ah Yusuf, Muhammad Yulianto Listiawan, Makhfudli Makhfudli

**Affiliations:** 1 Faculty of Nursing, Airlangga University, Surabaya, Indonesia; 2 Faculty of Vocational, Airlangga University, Surabaya, Indonesia; 3 Faculty of Medicine, Airlangga University, Surabaya, Indonesia; University of Brasilia, BRAZIL

## Abstract

**Background:**

Family involvement in overcoming the severity of leprosy is very important in the life of leprosy sufferers in communities who experience the clinical and, psychological, social and behavioral consequences of the disease. However, this need, psychosocial, is felt to be not optimal. This study is to identify how the experiences of family members as caregivers provide assistance to individuals with leprosy in improving healing and maintaining patterns of interaction in the family.

**Methods:**

The design uses qualitative research with in-depth, face-to-face interviews with family members in a semi-structured manner with the hope of obtaining complete data. Using purposive sampling with Participatory Interpretative Phenomenology analysis, there are 12 families with 15 family members consisting of 4 men and 11 women.

**Results:**

This study produced a family theme that tried to follow what would happen to individuals with leprosy, with four sub-categories: 1) Using various coping alternatives to recognize the disease, 2) Family members in the shadow of leprosy, 3) Trying to empathize with other family members. sick, 4) Caring for the emotional response of the family and seeking support.

**Conclusions:**

This analysis shows that deficiency in cognitive aspects can be closed by maintaining a lifestyle in the family through efforts to understand, support, establish communication, increase maximum involvement in restoring self-confidence, especially in individuals with leprosy with psychosocial problems in the family. The results of this study can be used as psychosocial support in maintaining communication between family members to support treatment programs and accelerate the recovery of leprosy.

## Author summary

Although families have high empathy for the physical, psychological, and social problems faced by individuals with leprosy, they are rarely involved in the personal affairs of individuals with leprosy, and there are indications that they left them to cure their disease. Meanwhile, overcoming problems in their own way without involving other family members, becomes the main choice for people with leprosy to avoid conflicts between family members, because they realize that leprosy is a disgrace in their family. However, the interaction between family members is an Indonesian culture that continues to be preserved. And for whatever reason, the culture of interaction between family members cannot be abandoned, even though there are obstacles that must be faced because this is a tool to strengthen kinship. The writer found that the family tried to help in healing leprosy suffered by their family members, even though they were in the shadow of leprosy. Through various coping strategies implemented, families try to help the healing process of their illness, with the hope that they will rise from adversity and have high motivation to recover. And restoring the confidence of people with leprosy is important to support a definitive treatment program.

## Introduction

The risk of leprosy transmission has become a serious threat in the world, with the emergence of new cases in children by 7.4%, of which 72.17% have level 2 disability, and Indonesia contributes to the discovery of new cases of leprosy in children by 13.41%., of whom have level 2 disability by 55.80% [1]. This indicates that, in Indonesia, the family environment is a source of leprosy transmission so that the main priority to break the chain of transmission and the healing process of leprosy in the family is important, although in Indonesia there have been no reports regarding the transmission. However, in India, based on the results of research on the transmission of leprosy in family clusters, as many as 5.44% are dominated by men, and the majority (90%) cases of children have family members suffering from lepromatous leprosy [2].

Related to the risk of this severity, maintaining a pattern of interaction between family members is very necessary to support efforts to cure and prevent leprosy disability [3,4]. But the atmosphere has been hampered by the attitude of the family, who are still struggling to deal with emotional responses related to traumatic events, sadness, feelings of shame, confusion, where these elements are also faced by people with leprosy [5]. Some literature reviews find the attitude of family members who are vulnerable to disharmony in the family, including teens show an excessive fear response to contracting leprosy [6], and for women, the psychological burden is felt far more painful [7–10], and families trying to hide their illness from their family environment to protect them [11].

Stigma-prone families still consider accepting the presence of individuals with leprosy in the family [12]. Meanwhile, children who enter adolescence still need modification of mind and behavior [13]. This is a very difficult test to be able to communicate and accept the existence of individuals with leprosy who create stress in the family. Because leprosy is always connected with stigma [14–18], families and sufferers are always in the shadow of psychiatric morbidity [19] and are faced with physical, psychological, and social problems [20], which can negatively impact the process of family life [21]. That is evidence that family members living with leprosy are very at risk of getting a label of “leprosy family “by the community environment, more so that sufferers suffer permanent disability due to leprosy [22], however, still there are some family members with leprosy sufferers who have permanent disability not affected [23].

Besides, leprosy experienced by family members is exacerbated by other factors that affect the mind of leprosy sufferers in the family, such as individuals with leprosy feel neglected [24–26], illness with God’s curse and God’s retribution for his actions and sins [27,28], difficult to treat, diseases that are severe [29], very susceptible to children [30], and the community feels disgusted to get along [28], so they do not want to get close [17], which sometimes causes disharmony in the family because it is considered an embarrassing disease [31]. And slowly, leprosy suffered by one family member can break communication between family members [32], and will unexpectedly break the kinship between other family members [33]. As a member of the family, some individuals with leprosy have thoughts of concern for the mentality of family members, they are afraid to discuss their problems with their family because of the risk of the family being embarrassed because they were found to have leprosy, and therefore at the beginning of diagnosis, they hid their illnesses [34], even though they have the motivation to recover and struggle to avoid disability [35].

Sharing information about leprosy and the risk of leprosy to family members based on the results of a systemic review of research can increase mutual understanding among family members [36], while the results of a literature review of several studies indicate that family interventions can reduce mental burdens and strengthen family resilience to face discrimination [37]. These interventions include psychoeducation [38], individual and family counseling [39,40], forming self-care groups [41], and group discussions between lay people and peer counselors [42].

Involving family members in the care and treatment of people affected by leprosy is relatively new where the attention that will be given to people with leprosy takes years. Support from the family in such situations can vary, depending on the initiative of the family, the sufferer himself, the opinions of other family members, and health professionals. The initiative to be able to discuss the problem of leprosy with other family members becomes very sensitive to be discussed, as it is done by health workers because of the risk of experiencing fear, anxiety, or stigma [43]. Family members may blame each other for leprosy in the family or family members do not want to come close when they find out that one of their family members has leprosy, and thus they do omission and do not want to treat[32], but that too can make families aware of the importance of their presence to provide social support in improving motivation to heal.

Research on interventions in families with one family member suffering from leprosy, so far limited to efforts to reduce stigma by providing support to children and from other families or relatives of parents who have leprosy [44], but the lack of this research shows that there are no interventions carried out in a natural context and trials to assess family members, and the results achieved from other families in the perspective of active family involvement address the risk of severity due to leprosy in the family in long term.

### Aims

This study aims to explore the life experience of leprosy families in maintaining interaction patterns in the family to support healing in leprosy patients.

## Methods

### Ethics statement

Ethical approval for this study was obtained from the Regional Ethical Review Board (No. 086 / EC / KEPK—S2). Parents signed the consent form, and children were also asked whether they agreed before being included in the study. Participants were assured of the confidentiality and anonymity of their participation. The research was carried out in accordance with the Declaration of Helsinki [45].

### Research design

A qualitative phenomenological approach has been chosen to explore the life experiences of leprosy families in maintaining interaction patterns in the family to support healing in leprosy patients as a meaningful experience [46]. The study process followed the Consolidated Criteria for Reporting Qualitative Research (COREQ) Checklist and the consolidated criteria for tracing qualitative research. Besides, the COREQ strategy was used to report this research [47]. Researchers have explored to find reflections on important themes that describe the phenomena that occur in leprosy patients and their families using this guide [48], and through writing, researchers connected between categories that were event-oriented and paid attention to the balance of the research theme by looking at each part as a whole. Furthermore, through the inductive approach method, thematic analysis was carried out [49].

### Participants

Participants were recruited through purposive sampling technique, the characteristics of age, gender, the status of members in the family, education level, were considered in sampling. A diverse and representative sample that reflected the population of family members was taken to get meaningful perceptions and experiences. Individual criteria in this study have been determined, namely (1) families with family members suffering from leprosy who lived in one house (2) family members who had kinship with people with leprosy. Data saturation was used to determine the number of samples, and data saturation was reached at the 13^th^ interview because no new information emerged [50]. Furthermore, two more participants were interviewed to check the new information. Data saturation and the final sample were then confirmed in the 15^th^ participant.

### Data collection

Researchers conducted in-depth face-to-face interviews with leprosy patients and/or family members in a semi-structured manner in the hope of obtaining complete data. Interviews were conducted individually to get a deeper understanding of the experience that matches what the researchers were researching [51]. The researchers invited families and people with leprosy to attend in a special room that has been provided at four Community Health Centers in Gresik Regency, Indonesia, and one by one the participants were interviewed. Before the interview was started, written consent and socio-demographic data were collected, and then, the researchers verbally asked for permission to record the voice of the conversation using a voice recorder that had previously been checked for accuracy of the recording device. Interviews were conducted starting from March–April 2021 which were attended by researchers and participants only, lasted between 40 and 60 minutes. Thematic interview guides with open-ended questions were developed, which were used by researchers, including the opening question “What do you feel (leprosy patients, children, wife, husband, nephew) with leprosy in the family?”. Data collection was carried out by two researchers. Ah yusuf is a Professor in the field of nursing with an interest in mental nursing with a concentration on psychosocial problems and is currently the dean of the Faculty of Nursing, Airlangga University. Abd. Nasir is a PhD candidate in nursing at Airlangga University, and currently a lecturer in psychiatric nursing with an interest in psychosocial issues in which he has produced two qualitative studies on psychosocial problems in chronic illness. All participants were interviewed and observed for one round by the researchers directly, using tools in the form of field notes and data recording devices. However, the researchers have also made clarifications to the participants again because there are some data that require further explanation. The interview guide was used to remind researchers about the topic of discussion and ensure that all major topics have been discussed, including a discussion of the life experiences of leprosy families in maintaining interaction patterns in the family to support healing in leprosy patients. Individual interview results were transcribed and confronted with nonverbal responses through field notes for data analysis and review to improve data accuracy [50].

### Data analysis

All interviews were transcribed verbatim and labeled with participant code. Interviewers simultaneously transcribed all data, allowing for consistent reflection and exploration of new ideas. The transcription was adapted to Indonesian and matched against the audio recording to ensure accuracy. Data analysis used Interpretative Phenomenological Analysis approach [52]. Following the data analysis guide of Smith et.al, 2009 (Table 1), interview transcripts and field notes were read carefully to identify emerging themes, then the researchers also categorized important related words [53].

**Table 1:** Phenomenological Analytic Process.

Smith’s steps	Application in this Paper
Reading and Re-reading	The researchers read it over and over again until the researchers got an understanding of the position of the important words.
Initial Noting	The researchers analyzed the contextual meaning of the words through searching from various sources to get the real meaning of the word.
Developing Emergent Themes	The researchers analyzed sentences to make formulations into sub-themes and important themes. Furthermore, the researchers reflected the sentences into sub-themes and themes.
Searching for connection of cross emergent themes	The researchers linked themes to one another by making a sequential scheme so that it appears a theme that is intertwined and related.
Moving the next cases	The analysis of the next participant up to the last participant was done according to the principle based on stages 1–4
Looking for patterns across cases	The researchers looked for patterns that emerge among participants. From these patterns, the researchers formulate them into themes
Taking interpretations to deeper levels	The researchers did a deeper and more interpretive analysis to find out the original meaning

The researchers read the text as a whole and tried to understand the overall meaning and developed keywords and concepts through dialogue with the text. Researchers also maintained openness through reflection efforts on various interpretations to monitor assumptions and biases through the triangulation process with participants [50]. Furthermore, themes were interpreted from the components of the experience to the whole experience and back again. Researchers sought to gain understanding and engagement with texts related to the phenomenon under study. Finally, each sentence was analyzed in which it was confronted with the data in the field notes, and through this process, important themes were found. These themes were then reconstructed into a description of the life experiences of leprosy families in maintaining interaction patterns within the family to support healing in leprosy sufferers. The validity of this study has been evaluated using the concepts of credibility, confirmability, dependability, and transferability. Some of the Examples of Relevant Questions are in Table 2.

**Table 2:** Example of Relevant Questions.

• Since when have you been feeling the symptoms of leprosy, do other family members know about this disease? • How do the spouses and other family members respond to leprosy? • How about the environment around you, what do you feel? • What are your expectations in family life towards family members and the environment around you, what do you do?

## Results

### Participants demographic profile

A total of 12 families with 15 family members participated in this study, consisting of children, wives, husbands, and nephews with an age range of 16–65 years consisting of 4 boys and 11 girls. Family members and their participation in the study are presented in Table 3

**Table 3:** Family members and their participation in the study.

Family composition	the status of an individual with leprosy in the family	Age of individual with leprosy	Type of leprosys	Grade of disability	Family interview participants	Interview with individual with leprosy	Interview with partner	Interview with child
Family 1: mother, father, 1 daughters, 2 son	Father	52	Multi Bacillary	2	Mother and daughter		Mother (48 years)	daughter (16 years)
Family 2: Mother, father, 2 daughters	Father	55	Multi Bacillary	2	Father	Father (55 years)		
Family 3: Nephew, Niece, 2 sons, 2 daughters	Son	20	Multi Bacillary	2	Father, Mother		Nephew (50 year) Niece (45 years)	
Family 4: mother, 2 son, 1 daughter	Mother	60	Multi Bacillary	1	Mother	Mother (60 years)		
Family 5: Mother, 1 sons, 2 daughter	Mother	32	Pauci Bacillary	1	Father	Father (35 years)		
Family 6: mother, father, 1 son	Father	55	Multi Bacillary	2	Mother, Son		Mother (48 years)	Son (26 years)
Family 7: Mother, father, 2 daughter	Father	45	Multi Bacillary	2	Mother		Mother (40 years)	
Family 8: Mother, father, 2 sons	Father	52	Multi Bacillary	2	Mother		Mother (48 years	
Family 9: mother, 1 daughters	Mother	70	Multi Bacillary	2	Daughter			Daughter (50)
Family 10: Father, Mother, 1 sons, 1 daughter	Mother	44	Multi Bacillary	1	Mother	Mother (44 years)		
Family 11: Father, Mother, 2 sons, 1 daughter	Mother	49	Pauci Bacillary	1	Mother	Mother (49 years)		
Family 12: Father, Mother, 1 daughter	Mother	26	Multi Bacillary	2	Mother	Mother (26 tahun)		

### Theme

In general, the theme that emerges from the data is that trying to follow the pattern of life that occurs in the family. This theme summarizes the behavior of the family in supporting individuals with leprosy to overcome the risk of severity in the context of interactions among family members. The completed data is presented in Table 4.

**Table 4:** Themes, and Subthemes.

Themes	Subthemes
Trying to recognize leprosy from applied assumptions	1. Using feelings to convince assumptions
	2. Interpreting information from a credible source to justify assumptions
Family members under the shadow of leprosy	1. Stereotypes and fear of social sanctions
	2. Fantasy of worrying conditions and desire to be filial
Seeking empathy to sick family members	1. Trying to understand the mental and emotional state
	2. Understanding and worrying about future situations
	3. Seeking alibis and distracting to avoid attacks of stigma and discrimination
Caring about the emotional responses of the family and trying to find support to avoid conflicts in the family	1. Trying to protect the disease to maintain communication among family members
	2. Seeking treatment without the involvement of many people

### Trying to recognize leprosy from applied assumptions

So far, families only used estimates to confirm the characteristics of leprosy. And, it becomes clear after getting an understanding from health workers.

#### Using feelings to convince assumptions

Leprosy is always disguised as other skin diseases so that when leprosy occurs, it is difficult to distinguish between leprosy and other skin diseases. Some parents experience misperceptions, as expressed by the mother of an individual with leprosy.

“I think it is a common skin disease, and my child also never complains about anything until he graduates from high school. Even though, he has been having tinea versicolor since 4 years ago.” (mother, 45 years old).

It is the same with a disease experienced by men suffering from leprosy. The wrong interpretation of the perception of leprosy can facilitate the emergence of maladaptive coping so that they feel guilty. The following is the statement:

“I think it is just common flu, sir because there are also a few colds. But I see that my children look confused because the flu is not getting better until my husband has difficulty in moving his hands. I immediately take him to the health service." (a wife, 45 years old,).

Other informants revealed that due to their ignorance of identifying the disease, they regretted being late in getting definitive treatment.

“… I say to him (husband), it is just tinea versicolor. Later it is given Kalpanak medicine so that it can heal … I was shocked when I was told by the health officer, he said that it is leprosy” (a wife, 48 years).

#### Interpreting information from a credible source to justify assumptions

Some family members were worried about the condition of their parents’ illnesses, the statement is as follows:

“I am confused about what my mother’s disease is, how come it does not heal with the various skin medicine that I give.” (daughter, 30 years old).

Other informants also felt curious and wanted to try to find justification, because of their experience during seeking treatment. But the result was that they had not yet received clear information about the type of illness of their family. The following is what expressed by the child of a mother was:

“I am very curious about what revealed by a private practicing doctor was that he did not explain what my mother’s illness was. He only said that the medicine for my mother’s disease was only available at the community health center.” (40 years old daughter).

### Family members under the shadow of leprosy

The second finding describes the description of family members about the difficulty in escaping from the image of leprosy so that there is a desire to pay attention as evidence to be devoted to individuals with leprosy.

#### Stereotypes and fear of social sanctions

The family was very worried when a an individual with leprosy was outside the house. One of the wives of an individual with leprosy revealed:

“In my heart, I am very worried if the illness of my husband is discovered by many people later … I do not know what happens in the family if the illness of my husband is discovered by many people.” (wife, 48 years old)

Families have predicted that stigma and discrimination will become an inseparable part of their personal and family lives. One of the nephews expressed his concern:

“It is clear that later my family will be gossiped by neighbors … I am afraid that the sale will not be sold if people find out about my nephew’s disease” (A nephew, 50 years).

#### The fantasy of worrying conditions and desire to be filial

Their children believed that this was the best time to serve and the right way to show the children’s devotion to their parents, so they gave up their chances to get married. A child said:

“Every day I always take care of my father, even though I am working … I want to show the devotion of a son to parents.” (26 years old boy)

For wives who felt that their husbands had lost their jobs, they tried to change their social roles for their survival. A wife of a husband who suffers from leprosy said:

“Every day I work in the fields to make a living and I ask my husband to just rest at home. I feel sorry for him when he meets people he will be embarrassed because his skin is like crocodile skin.” (a wife, 40 years old).

### Seeking empathy to sick family members

This finding identifies how the responses of family members can psychologically support family members suffering from leprosy so they do not get discriminatory treatment from the surrounding community.

#### Trying to understand the mental and emotional state

Everyone around the person suffering from leprosy is very careful and aware of the mood of family members with leprosy and adjusts themselves so that there is no conflict. Family members know how to convey something to an individual with leprosy. One child of a leprosy parent revealed:

“I really feel the condition of my mother at this time that she is very sad with her condition … I am very sorry for my mother” (a daughter, 54 years).

Children of other sufferers also said:

“When I first knew that my father came from wanderings with a face with creepy skin, I screamed. And, until now, when I need something, I say to my mother, because I feel sorry for my father who does not work” (14-year-old daughter)

Meanwhile, one couple tried to calm his wife who was upset and very scared due to leprosy.

“Never mind ma’am (wife). You do not have to think about the disease, it will get better. You don’t have to worry, you have already been given medicine by the doctor.” (a husband, 45 years)

#### Understanding and worrying about future situations

A mother who is entrusted by a relative to care for a child suffering from leprosy expressed her concern for her nephew’s condition. She was worried about her future and was proud of the efforts of her nephew. She said:

“I really feel sorry for my nephew, she is still young. He has no normal body (very short). And, she was given a disease like this (leprosy) … until now she has no partner and has no intention of looking for a partner."(aunt, 45 years).

There were also couples’ families who already knew the disease and tried to calm their partners so they did not get confused and stressed.

“Never mind dear(wife), later when you take your medicine, the spotting on your face will be thin and will disappear by itself. Don’t be afraid, it’s a pity that your child is still a baby.” (a husband, 30 years)

#### Seeking alibis and distracting to avoid attacks of stigma and discrimination

The way that husbands do so that their wives are not stigmatized by the community for suffering from leprosy is by disguising leprosy with other diseases and diverting public opinion towards discriminatory assumptions to avoid negative perceptions. One husband described his experience when he met his friend:

“I always tell people that my wife has a high level of drug allergy after taking the rheumatic medicine so that it heals a little longer. And, as a result of the allergy treatment, her face is little black, but not bad.” (husband, 49 years old)

### Caring about the emotional responses of the family and trying to find support to avoid conflicts in the family

This last theme identifies the efforts made by persons suffering from leprosy to solve their own problems without involving other family members so that they did not become a burden and a source of problems in the family due to their illness. They also beg other family members to maintain a mutually satisfying relationship.

#### Trying to protect the disease to maintain communication among family members

Persons with leprosy feel that they are part of the person who brings shame to the family. They also know that the risks that they receive affect the relationship between family members and the community because the stigma of leprosy exceeds the disease. Thus, motivating them to seek support so that there is no conflict in the family. one of the sufferers said:

“I want to recover without being noticed by my husband. The spots on my hands are covered by always wearing long-sleeved clothes and if I take medicine (leprosy medicine), I always take it by myself and make appointments with officers outside the health service hours." (a woman with leprosy, 44 years old)

Other informants used other rational reasons to seek treatment without causing disharmony in the family. They knew the consequences regarding the peace among family members. If the disease is known by all family members, especially their children, it will cause serious burdens in the association.

“Only me and his father (husband) who know about this disease (leprosy) without known by my teenage son. I am afraid that my teenage son will know and be ashamed of his friends because his mother is suffering from this disease (leprosy).” (a woman with leprosy, 49 years old)

#### Seeking treatment without the involvement of many people

The best solution for maintaining relationships between family members is by not involving other people in the treatment process. Some sufferers realize the importance of undergoing treatment, even if it takes a long time. This is what an individual with leprosy said:

“When it is time to check up and take medicine, I will immediately go to the health service” (a woman with leprosy. 26 years old)

Some individuals with leprosy are greatly helped by the services provided by health workers. They realize how important health workers are for the healing process for their disease, other individuals with leprosy revealed:

“Fortunately, there is this woman (a leprosy program holder at the Community Health Center). The person is very attentive. If my brother does not take the medicine, he will definitely deliver the medicine, and sometimes it is given to the health worker who is in the village and I take it, (a woman with leprosy, 45 years old)

## Discussion

This study, based on a family-centered approach in Indonesia, in which the Indonesian people consider leprosy to be a disgrace in the family and spiritually religious, some Indonesians consider leprosy to be a curse from God [54]. This study aims to understand their experience in maintaining interaction patterns in the family to support healing in leprosy patients. Below, the researchers describe a new contribution, which complements the results of previous research on this topic. This study, unlike previous studies, has highlighted the inevitable and profound impact that leprosy patients and their families have on leprosy in the family. It emphasizes how the impact of physical problems due to leprosy can affect all family members, especially regarding the perceived stigma and discrimination, the support provided, the relationship between family members, their relationship with the closest environment, their social, and work life.

Likewise, other studies have identified that stigma is one of the dominant factors for several problems in individuals with leprosy, such as social restrictions [55], life [14,55], individual coping [31,56], psychological well-being of individuals with leprosy [57], family restrictions on them [58], and family toughness [37]. Other findings also indicate that the physical and social impacts have been experienced continuously by families that affect their overall psychological well-being, and this is an additional feature of their experiences when living with individuals with leprosy [28].

However, several studies that focus on family involvement in suppressing the risks posed have been proven effective for patients with various other chronic diseases, including to improve self-care [59], medication adherence [60–62], improve self-efficacy [63], disease control [64], reduce stress [65], ease family burdens [66], and improve relationships [67,68]. Apart from these benefits, this research has found the acquisition of new habits from the family (replacing family social roles, family social support, understanding emotional responses, putting family emotions into the emotions of sick members, and direct involvement in care), which can bring big changes in patients’ habits into family social life that were previously difficult for them and only produced difficult responses.

This research as in other studies has discussed chronic diseases in the family, that chronic diseases in the family can direct family members to understand them and change their attitudes and think that now there is one family member who has the disease with serious attention needs [69].

However, the results of this study indicate that leprosy sufferers and their families are only open with health workers and certain family members and are closed to other families and the surrounding community, and try to find alternative solutions themselves outside the definitive treatment program so that the condition of illness becomes peaked with the onset of leprosy reaction as the beginning of permanent disability [70–73]. This is an unfavorable impact on patients and their families that must be anticipated, and in this sense, several studies have discussed the positive consequences received by families and patients on adaptive coping [73].

Through effective communication applications in disease management, leprosy patients are able to show good self-concepts [74–76], reduce negative emotional responses [77], high motivation to recover [78], open about her illness [42], increase self-confidence [79], and other family members are able to provide adequate care assistance [38,76].

The most interesting finding of this research is that people with leprosy want to be a perfect person in the household, that is, to live physically and mentally prosperous with their spouse, and the environment around them also accepts their existence. This topic is aligned with the way they seek to guarantee a prosperous life because psychological needs are met as adults. However, some research results, do not support their desires and their perceptions tend to be negative for individuals with leprosy [80,81], and even lower than tuberculosis [82].

In contrast to other chronic illness studies on psychological well-being, which discuss the importance of securing a prosperous life from spouses and families, they get extraordinary support from their families through the adaptation of new life experiences [83,84]. Besides, the researchers also found that leprosy patients want to be accepted by families and the community like normal people in general. In this sense, individuals with leprosy are always in the shadow of community stigma [28],[85] which are equally experienced by AIDS sufferers [86], and in accordance with this research, due to the severity of the perceived stigma, sufferers and spouses do not want to be open with their children and the surrounding community, and only want to discuss the problem with officers only.

Living with a person suffering from leprosy is felt differently by his family [26], especially for adolescents [6] and based on the results of the Systematic Literature Review, this feeling will increase suffering for patients and family [44] and reduce family self-esteem [11,33]. Although the family has acted to overcome the illnesses and changes that occur in patients to recover quickly, it is also necessary to improve life control in improving self-concept as reported by previous studies [87]. It is very important that mental health nurses must be present when individuals with leprosy and their families experience complicated situations and help with the problem [74,87], due to the dimension of the relationship between nurses and patients/family is one of the fundamental aspects of client care with psychosocial problems [16]. With his presence, nurses can explore the feelings faced by family and discuss mental-emotional problems to connect internal conditions to their external environment, and at that time nurses must be able to encourage families and communities to improve self-care abilities [88] and can provide social support for people suffering from leprosy through support groups created [89].

### Relevance for nursing practice

Other family members cannot be expected to be maximally involved in the healing process because they have their respective responsibilities in which there is a moral responsibility to get involved as an implementation of family duties to care for sick family members, especially in chronic illnesses [90], in addition to acting as a liaison with health workers, especially nurses [91–93]. As shown in analysis and identification in other studies, families can still be involved in reflecting in a critical of their understanding and the way they provide assistance in overcoming problems faced by sufferers with chronic diseases [94] such as leprosy [11,33]. Thus, maintaining the pattern of life in the family to support the physical and emotional well-being of individuals with leprosy is very important in the life of individuals with leprosy. Through the community mental health nursing application, family involvement is an important part of providing mental nursing care, especially psychosocial problems with chronic disease problems such as leprosy [95]. Besides, increasing awareness of the potential importance of maintaining a pattern of life in a family with a family member suffering from leprosy can increase maximum involvement and self-confidence to recover through adaptive coping strategies [76] and can be used as psychosocial support in maintaining communication between family members to support treatment programs and accelerate the recovery of leprosy.

### Study limitations

The participants in this study feel comfortable expressing their experiences in dealing with the risk of leprosy in the family, but there are different characteristics, maybe their perceptions are also different. Besides, because leprosy is a disgrace to other families, they are very careful in expressing their feelings, and the way they express their feelings is also different. Although both of them pose potential limitations, with the therapeutic communication approach, these two limitations are not a barrier to obtaining natural and complete data. Furthermore, the sampling used a purposive sample from families of people suffering from leprosy in rural areas. This may be a slightly different perception of leprosy sufferers in urban areas, although they all agree that leprosy is a disgrace to their families. The analysis is based on the data that has been found, but triangulation with experts and leprosy program holders may contribute much more to their knowledge and perceptions through a professional approach, including with affected families.

## Conclusion

This research is in the context of the family to improve understanding of family life in maintaining a harmonious relationship between family members due to leprosy in the family. The high initiative to heal by leprosy sufferers, makes their families feel comfortable and does not feel pressured, so they are motivated to help the healing process of their disease. This action becomes a core element in promoting anticipation of the risk of severity of leprosy patients oriented towards family life. This is to improve the ability of interaction patterns and provide mutual support physically, psychologically, and socially for people with leprosy. This strategy aims to increase family awareness in supporting individuals with leprosy to avoid the risk of severity

## Supporting information

S1 COREQ Checklist The Consolidated Criteria for Reporting Qualitative Research (COREQ) checklist. (DOCX)

S1 Verbatim Results of in-depth face-to-face interviews. (DOCX)
