# Correction: The life experience of leprosy families in maintaining interaction patterns in the family to support healing in leprosy patients in Indonesian society. A phenomenological qualitative study

**DOI:** 10.1371/journal.pntd.0010951

**Published:** 2022-12-01

**Authors:** The PLOS Neglected Tropical Diseases Staff

## Notice of republication

This article was republished on November 9, 2022 to address a terminology issue that came to light after the article’s initial publication: the term “leper” was removed from the article and replaced by “individuals with leprosy” or “individual with leprosy.” We understand that the removed term has negative connotations and has been discussed as contributing to stigmatization of individuals harboring this disease. The *PLOS Neglected Tropical Diseases* journal does not condone the use of the term “leper” in describing individuals with leprosy. We apologize to any readers who were negatively affected by the use of this term in the original versions of this article.

The rest of the content remains unchanged in the version of record (listed on the article’s webpage as Version 2) for the republished article.

The uncorrected proof (Version 1) was removed from the article’s *PLOS Neglected Tropical Diseases* webpage at the time of republication.
