# Supplementary material for: The life experience of leprosy families in maintaining interaction patterns in the family to support healing in leprosy patients in Indonesian society. A phenomenological qualitative study
Source: PLoS Negl Trop Dis. 2022 Apr 8;16(4):e0010264. doi: 10.1371/journal.pntd.0010264 (PMC9020682; doi:10.1371/journal.pntd.0010264)
Supplement: S1 COREQ Checklist — (DOCX) [file pntd.0010264.s001.docx]

The Consolidated Criteria for Reporting Qualitative Research (COREQ) checklist

centang (√)

| Cheklish | Options | |
| --- | --- | --- |
|  | Yes | No |
| **Aim** |  |  |
| Is the research question a relevant issue? | √ |  |
| Is the aim sufficiently focused, and stated clearly? | √ |  |
| Does the title of the article give a clear account of the aim? | √ |  |
| **Reflexivity** |  |  |
| Are the researcher's motives, background, perspectives, and preliminary hypotheses presented, and is the effect of these issues sufficiently dealt with? | √ |  |
| **Method and design** |  |  |
| Are qualitative research methods suitable for exploration of the research question? | √ |  |
| Has the best method been chosen with respect to the research question? | √ |  |
| **Data collection and sampling** |  |  |
| Is the strategy for data collection clearly stated (usually purposive or theoretical, usually not random or representative)? | √ |  |
| Are the reasons for this choice stated? | √ |  |
| Has the best approach been chosen, in view of the research question? | √ |  |
| Are the consequences of the chosen strategy discussed and compared with other options? | √ |  |
| Are the characteristics of the sample presented in enough depth to understand the study site and context? | √ |  |
| **Theoretical Framework** |  |  |
| Are the perspectives and ideas used for data interpretation presented? | √ |  |
| Is the framework adequate, in view of the aim of the study? | √ |  |
| Does the author account for the role given to the theoretical framework during analysis? | √ |  |
| **Analysis** |  |  |
| Are the principles and procedures for data organization and analysis fully desribed, allowing the reader to understand what happened to the raw material to arrive at the results? | √ |  |
| Were the various categories identified from theory or preconceptions in advance, or were they developed from the data? | √ |  |
| Which principles were followed to organize the presentation of findings? | √ |  |
| Are strategies used to validate results presented, such as cross-checks for rivalling explanations, member checks, or triangulation?  If such strategies are not described in this section, they should appear as validity discussion later in the report | √ |  |
| **Findings** |  |  |
| Are the findings relevant with respect to the aim of the study? | √ |  |
| Do they provide new insight? | √ |  |
| Is the presentation of the findings well organized and best suited to ensure that findings are drawn from systematic analysis of material, rather than from preconceptions? | √ |  |
| Are quotes used adequately to support and enrich the researcher's synopsis of the patterns identified by systematic analysis? | √ |  |
| **Discussion** |  |  |
| Are questions about internal validity (what the study is actually about), external validity (to what other settings the findings or notions can be applied), and reflexivity (the effects of the researcher on processes, interpretations, findings, and conclusions) addressed? | √ |  |
| Has the design been scrutinized? | √ |  |
| Are the shortcomings accounted for and discussed, without denying the responsiblity for choices taken? | √ |  |
| Have the findings been compared with apprpriate theoretical and empirical references? | √ |  |
| Are a few clear consequences of the study proposed? | √ |  |
| **Presentation** |  |  |
| Is the report easy to understand and clearly contextualized? | √ |  |
| Is it possible to distinguish between the voices of the informants and those of the researcher? | √ |  |
| **References** |  |  |
| Are important and specific sources in the field covered, and have they been appropriatedly presented and applied in the text? | √ |  |
