# Supplementary material for: The life experience of leprosy families in maintaining interaction patterns in the family to support healing in leprosy patients in Indonesian society. A phenomenological qualitative study
Source: PLoS Negl Trop Dis. 2022 Apr 8;16(4):e0010264. doi: 10.1371/journal.pntd.0010264 (PMC9020682; doi:10.1371/journal.pntd.0010264)
Supplement: S1 Verbatim — (DOCX) [file pntd.0010264.s002.docx]

***Trying to recognize leprosy from applied assumptions***

So far, families only used estimates to confirm the characteristics of leprosy. And, it becomes clear after getting an understanding from health workers.

***Using feelings to convince assumptions.*** Leprosy is always disguised as other skin diseases so that when leprosy occurs, it is difficult to distinguish between leprosy and other skin diseases. Some parents experience misperceptions, as expressed by the mother of a leper.

"I think it is a common skin disease, and my child also never complains about anything until he graduates from high school. Even though, he has been having tinea versicolor since 4 years ago.” (mother, 45 years old).

It is the same with a disease experienced by men suffering from leprosy. The wrong interpretation of the perception of leprosy can facilitate the emergence of maladaptive coping so that they feel guilty. The following is the statement:

“I think it is just common flu, sir because there are also a few colds. But I see that my children look confused because the flu is not getting better until my husband has difficulty in moving his hands. I immediately take him to the health service." (a wife, 45 years old,).

Other informants revealed that due to their ignorance of identifying the disease, they regretted being late in getting definitive treatment.

"... I say to him (husband), it is just tinea versicolor. Later it is given Kalpanak medicine so that it can heal ... I was shocked when I was told by the health officer, he said that it is leprosy" (a wife, 48 years).

***Interpreting information from a credible source to justify assumptions.*** Some family members were worried about the condition of their parents' illnesses, the statement is as follows:

“I am confused about what my mother's disease is, how come it does not heal with the various skin medicine that I give.” (daughter, 30 years old).

Other informants also felt curious and wanted to try to find justification, because of their experience during seeking treatment. But the result was that they had not yet received clear information about the type of illness of their family. The following is what expressed by the child of a mother was:

“I am very curious about what revealed by a private practicing doctor was that he did not explain what my mother's illness was. He only said that the medicine for my mother's disease was only available at the community health center.” (40 years old daughter).

***Family members under the shadow of leprosy***

The second finding describes the description of family members about the difficulty in escaping from the image of leprosy so that there is a desire to pay attention as evidence to be devoted to lepers.

***Stereotypes and fear of social sanctions.*** The family was very worried when a leper was outside the house. One of the wives of a leper revealed:

“In my heart, I am very worried if the illness of my husband is discovered by many people later … I do not know what happens in the family if the illness of my husband is discovered by many people.” (wife, 48 years old)

Families have predicted that stigma and discrimination will become an inseparable part of their personal and family lives. One of the nephews expressed his concern:

"It is clear that later my family will be gossiped by neighbors … I am afraid that the sale will not be sold if people find out about my nephew's disease" ( A nephew, 50 years).

***The fantasy of worrying conditions and desire to be filial.*** Their children believed that this was the best time to serve and the right way to show the children's devotion to their parents, so they gave up their chances to get married. A child said:

“Every day I always take care of my father, even though I am working … I want to show the devotion of a son to parents.” (26 years old boy)

For wives who felt that their husbands had lost their jobs, they tried to change their social roles for their survival. A wife of a husband who suffers from leprosy said:

"Every day I work in the fields to make a living and I ask my husband to just rest at home. I feel sorry for him when he meets people he will be embarrassed because his skin is like crocodile skin.” (a wife, 40 years old).

***Seeking empathy to sick family members***

This finding identifies how the responses of family members can psychologically support family members suffering from leprosy so they do not get discriminatory treatment from the surrounding community.

***Trying to understand the mental and emotional state.*** Everyone around the person suffering from leprosy is very careful and aware of the mood of family members with leprosy and adjusts themselves so that there is no conflict. Family members know how to convey something to a leper. One child of a leprosy parent revealed:

"I really feel the condition of my mother at this time that she is very sad with her condition ... I am very sorry for my mother” (a daughter, 54 years).

Children of other sufferers also said:

“When I first knew that my father came from wanderings with a face with creepy skin, I screamed. And, until now, when I need something, I say to my mother, because I feel sorry for my father who does not work” (14-year-old daughter)

Meanwhile, one couple tried to calm his wife who was upset and very scared due to leprosy.

"Never mind ma'am (wife). You do not have to think about the disease, it will get better. You don't have to worry, you have already been given medicine by the doctor.” (a husband, 45 years)

***Understanding and worrying about future situations.*** A mother who is entrusted by a relative to care for a child suffering from leprosy expressed her concern for her nephew's condition. She was worried about her future and was proud of the efforts of her nephew. She said:

“I really feel sorry for my nephew, she is still young. He has no normal body (very short). And, she was given a disease like this (leprosy)… until now she has no partner and has no intention of looking for a partner."(aunt, 45 years).

There were also couples' families who already knew the disease and tried to calm their partners so they did not get confused and stressed.

"Never mind dear(wife), later when you take your medicine, the spotting on your face will be thin and will disappear by itself. Don't be afraid, it's a pity that your child is still a baby.” (a husband, 30 years)

***Seeking alibis and distracting to avoid attacks of stigma and discrimination.*** The way that husbands do so that their wives are not stigmatized by the community for suffering from leprosy is by disguising leprosy with other diseases and diverting public opinion towards discriminatory assumptions to avoid negative perceptions. One husband described his experience when he met his friend:

“I always tell people that my wife has a high level of drug allergy after taking the rheumatic medicine so that it heals a little longer. And, as a result of the allergy treatment, her face is little black, but not bad.” (husband, 49 years old)

***Caring about the emotional responses of the family and trying to find support to avoid conflicts in the family***

This last theme identifies the efforts made by persons suffering from leprosy to solve their own problems without involving other family members so that they did not become a burden and a source of problems in the family due to their illness. They also beg other family members to maintain a mutually satisfying relationship.

***Trying to protect the disease to maintain communication among family members.*** Persons with leprosy feel that they are part of the person who brings shame to the family. They also know that the risks that they receive affect the relationship between family members and the community because the stigma of leprosy exceeds the disease. Thus, motivating them to seek support so that there is no conflict in the family. one of the sufferers said:

“I want to recover without being noticed by my husband. The spots on my hands are covered by always wearing long-sleeved clothes and if I take medicine (leprosy medicine), I always take it by myself and make appointments with officers outside the health service hours." (a woman with leprosy, 44 years old)

Other informants used other rational reasons to seek treatment without causing disharmony in the family. They knew the consequences regarding the peace among family members. If the disease is known by all family members, especially their children, it will cause serious burdens in the association.

"Only me and his father (husband) who know about this disease (leprosy) without known by my teenage son. I am afraid that my teenage son will know and be ashamed of his friends because his mother is suffering from this disease (leprosy).” (a woman with leprosy, 49 years old)

***Seeking treatment without the involvement of many people.*** The best solution for maintaining relationships between family members is by not involving other people in the treatment process. Some sufferers realize the importance of undergoing treatment, even if it takes a long time. This is what a leper said:

“When it is time to check up and take medicine, I will immediately go to the health service” (a woman with leprosy. 26 years old)

Some lepers are greatly helped by the services provided by health workers. They realize how important health workers are for the healing process for their disease, other lepers revealed:

"Fortunately, there is this woman (a leprosy program holder at the Community Health Center). The person is very attentive. If my brother does not take the medicine, he will definitely deliver the medicine, and sometimes it is given to the health worker who is in the village and I take it, (a woman with leprosy, 45 years old)
